# The combined use of raw and phylogenetically independent methods of outlier detection uncovers genome‐wide dynamics of local adaptation in a lizard

**DOI:** 10.1002/ece3.5872

**Published:** 2019-11-22

**Authors:** Alejandro Llanos‐Garrido, Javier Pérez‐Tris, José A. Díaz

**Affiliations:** ^1^ Informatics Group Faculty of Arts and Sciences Harvard University Cambridge MA USA; ^2^ Departamento de Biodiversidad Universidad Complutense de Madrid Madrid Spain

**Keywords:** ancestral variation, gene flow, genetic innovation, genotyping by sequencing, phenotypic convergence, *Psammodromus algirus*

## Abstract

Local adaptation is a dynamic process by which different allele combinations are selected in different populations at different times, and whose genetic signature can be inferred by genome‐wide outlier analyses. We combined gene flow estimates with two methods of outlier detection, one of them independent of population coancestry (CIOA) and the other one not (ROA), to identify genetic variants favored when ecology promotes phenotypic convergence. We analyzed genotyping‐by‐sequencing data from five populations of a lizard distributed over an environmentally heterogeneous range that has been changing since the split of eastern and western lineages ca. 3 mya. Overall, western lizards inhabit forest habitat and are unstriped, whereas eastern ones inhabit shrublands and are striped. However, one population (Lerma) has unstriped phenotype despite its eastern ancestry. The analysis of 73,291 SNPs confirmed the east–west division and identified nonoverlapping sets of outliers (12 identified by ROA and 9 by CIOA). ROA revealed ancestral adaptive variation in the uncovered outliers that were subject to divergent selection and differently fixed for eastern and western populations at the extremes of the environmental gradient. Interestingly, such variation was maintained in Lerma, where we found high levels of heterozygosity for ROA outliers, whereas CIOA uncovered innovative variants that were selected only there. Overall, it seems that both the maintenance of ancestral variation and asymmetric migration have counterbalanced adaptive lineage splitting in our model species. This scenario, which is likely promoted by a changing and heterogeneous environment, could hamper ecological speciation of locally adapted populations despite strong genetic structure between lineages.

## Graphical abstract

We combined gene flow estimates with different methods of outlier detection, to identify different sources of genetic variability in a lizard with deep genetic structure in the Iberian Peninsula. We found evidence of the maintenance of ancestral genetic variation and genetic innovations being favored when ecology demands adaptive convergence.

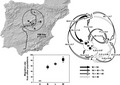

## 1 INTRODUCTION

Local adaptation, the process by which natural selection leads a population of organisms to be better suited to its particular environment than other populations of the same species, requires that different gene combinations are selected in different populations at different times during their evolutionary history (Endler, 1977; Hereford, 2009; Savolainen, Lascoux, & Merilä, 2013; Schluter, 2001; Wright, 1932). It is thus relevant to investigate the genetic footprint of past and present events that may shape adaptation as a dynamical process (Kokko et al., 2017). Such dynamism relies not only on how local adaptation interacts with current genetic dynamics, but also on how local adaptation has been favored, constrained, and/or blurred by the evolutionary history of populations (Laurent et al., 2016; McEntee, Tobias, Sheard, & Burleigh, 2018; Rosenblum, Hickerson, & Moritz, 2007; Rundle & Nosil, 2005). This temporal perspective can be provided by several genomic methods that in that respect supersede other approaches such as common garden or transplant experiments (Haasl & Payseur, 2016; Tiffin & Ross‐Ibarra, 2014).

It is possible to screen genome‐wide patterns of DNA polymorphism to detect locus‐specific signals of divergent selection by using theoretical predictions about the effects of positive natural selection on allele frequencies (Kim & Stephan, 2002; Luikart, England, Tallmon, Jordan, & Taberlet, 2003; Przeworski, 2002; Schlötterer, 2003). Such screening may reveal loci reflecting high levels of genetic differentiation between groups of individuals putatively subject to divergent selection (Han et al., 2017; Wu, 2001). However, the genetic underpinnings of adaptation may depend on the particular genetic background of populations, which have the potential to develop consistent phylogeographic patterns depending on how long they have been reproductively isolated from one another. Thus, the way in which populations locally adapt will depend on their phylogeographic history as realized in terms of past genetic change (either adaptive or stochastic, or influenced by gene flow; Barrett & Schluter, 2008; Han et al., 2017). This may often complicate the detection and interpretation of loci subject to selection (Rhode, Bester‐Van Der Merwe, & Roodt‐Wilding, 2017; Schield et al., 2017; Tigano, Shultz, Edwards, Robertson, & Friesen, 2017).

The most immediate approach to outlier analysis consists of uncovering which loci present traces of selection, and whether this selection is divergent or not (Tiffin & Ross‐Ibarra, 2014). To this end, Bayesian approaches that use logistic regression to partition *F*_ST_ coefficients into a population‐specific term and a locus‐specific term (e.g., Foll & Gaggiotti, 2008) have proved convenient. It is also possible to find outliers whose allele frequencies deviate from the expected under genetic drift alone, given the population tree (e.g., Bonhomme et al., 2010). For simplicity, we refer to these two approaches as raw outlier analysis (ROA) and coancestry‐independent outlier analysis (CIOA), respectively. If the two sets of outliers detected by these alternative approaches do not overlap, ROA and CIOA, combined with gene flow estimates, can shed light on the relative roles of ancestral genetic variation, admixture, and genetic innovations, as drivers of local adaptation dynamics (Barrett & Schluter, 2008; Haasl & Payseur, 2016). The lack of overlap between the outliers detected by ROA and CIOA could be meaningful in ecology‐driven phylogenetic structuring scenarios. This is because the ability of CIOA to detect SNPs related to the adaptive processes should be low when the relationship between ecology and genetic structure in the sample is strong (Bonhomme et al., 2010). In the limit, coancestry could be able to explain entirely the pattern of allele fixation. In these cases, such outliers should be detected more easily by ROA, while the ones underlying particular scenarios of local adaptation within the general branching pattern (independent to coancestry) would be more easily detected by CIOA (Haasl & Payseur, 2016).

**Figure 1:**
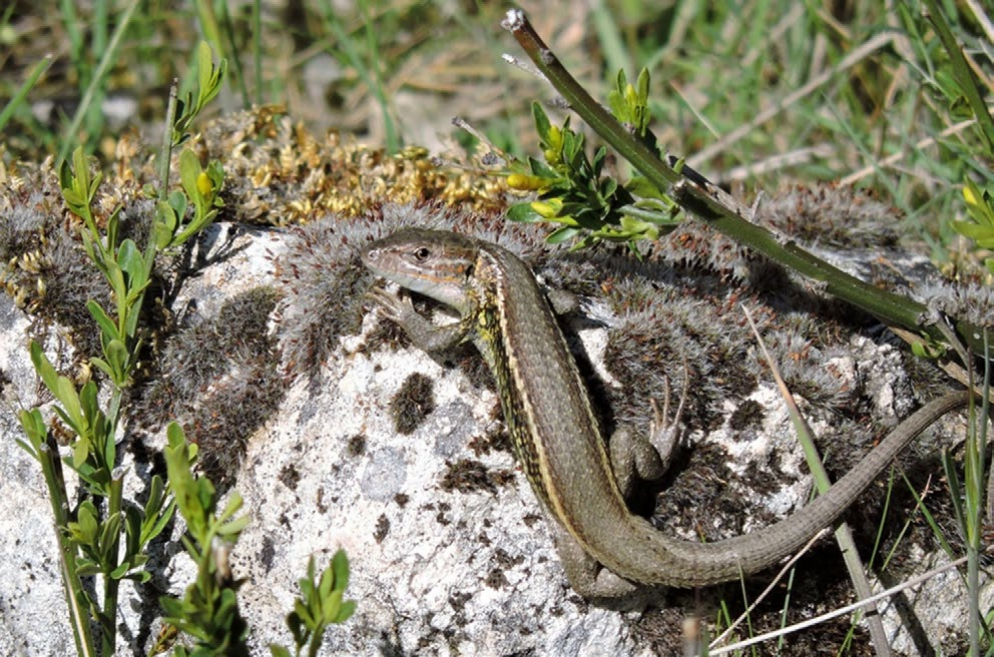
A male *Psammodromus algirus*, the model species of this study

We used genotyping by sequencing (GBS; Elshire et al., 2011) and combined ROA and CIOA to explore how the fixation of ancestral genetic variation and genetic innovation underlie convergent local adaptation. To do so, we used as model species the most abundant and widespread lizard in the Mediterranean region of the Iberian Peninsula: the large Psammodromus *Psammodromus algirus* (Díaz et al., 2017; Figure 1). This species shows consistent genetic and phenotypic divergence associated with environmental heterogeneity across its distribution range. The northwest part of its range is more humid and temperate than the southeast, and this is mirrored by broad changes in the vegetation patterns, with forests dominating in the west and more open spaces in the east (Díaz et al., 2017). The genetic diversity of *P. algirus* is structured in two mtDNA lineages, eastern and western, separated ca. 3–3.5 Ma (Carranza, Harris, Arnold, Batista, & Gonzalez De La Vega, 2006). Each mtDNA lineage typically shows a distinct phenotype for a heritable trait that could be adaptively linked to crypsis: lizards that inhabit the eastern, more open and grassier regions tend to display a striped dorsal pattern absent among western lizards, which inhabit more vegetated forests (Díaz et al., 2017). However, striped and unstriped phenotypes do not perfectly match the eastern–western lineage dichotomy. For instance, the population in Lerma that has been the focus of several previous studies (see Díaz, Pérez‐Tris, Tellería, Carbonell, & Santos, 2005; Santos, Díaz, Pérez‐tris, Carbonell, & Tellería, 2008; Santos, Pérez‐Tris, Carbonell, Tellería, & Díaz, 2009; Telleria et al., 2011) inhabits a forest archipelago that resembles the typical habitat of western lizards. The phenotype of these lizards is unstriped, fitting what we would expect if they were locally adapted to the forests they live in. However, the analysis of their mtDNA places Lerma within the E2 clade of the eastern lineage, which is almost exclusively composed of striped lizards (Díaz et al., 2017). Therefore, the Lerma population provides an excellent opportunity to investigate the sources of variation that can be locally selected to generate phenotypic convergence after long periods of genetic divergence.

First, ancestral genetic variation standing within lineages since before their divergence could allow populations to face high levels of environmental heterogeneity (i.e., adaptation by ancestral genetic diversity; Barrett & Schluter, 2008). This source of ancestral genetic variation should be detected by ROA in the form of divergent outliers with high levels of homozygosity in populations at the extremes of the phenotypic (and environmental) gradient, but with higher than average heterozygosity in the Lerma population. If this was the case, adaptive genetic variation would predate lineage splitting, when it could be either neutral, adaptive to an intermediate environment or maintained by adaptation to a geographically unstructured environmental heterogeneity (Huang, Tran, & Agrawal, 2016; Mackay, 1981; McDonald & Ayala, 1974; Paccard et al., 2018; Parter, Kashtan, & Alon, 2008; Welch & Jiggins, 2014). This variation would be divergently selected in the different ecological scenarios occupied by where eastern and western lineages (Díaz et al., 2017). Therefore, we would expect an overrepresentation of alternative alleles in lizards inhabiting each extreme of the environmental gradient (Nosil, Funk, & Ortiz‐Barrientos, 2009). Such overrepresentation should be consistent with the branching pattern of populations, since lineage splitting seemed to be ecologically driven (Díaz et al., 2017). However, these outliers should be less detectable by CIOA, because they would not show allele frequencies with enough deviation from the expected given the overall ecologically driven phylogenetic structure of populations. Second, local adaptation of populations faced with novel environments could have been attained through genetic innovation. Genetic innovation might be provided in Lerma by immigrants bringing genetic variants from populations already adapted to similar environments (i.e., adaptation by immigration; Peniston, Barfield, & Holt, 2019). Both eastern and western lineages would show highly divergent homozygosity not only in neutral loci but also in the ones that are under selection, because of both the maintenance of ancestral lineage splitting (genetic drift) and the adaptation to their respective extremes of an environmental gradient, respectively (Díaz et al., 2017; Räsänen & Hendry, 2008). Thus, if genetic innovation is the result of admixture, we also expect ROA to detect outliers with high heterozygosity, but not higher than the average heterozygosity of the entire genome. Finally, genetic innovation may also evolve from natural selection of locally arisen variants (Huber et al., 2015). In Lerma, such innovations should be uncovered by CIOA in the form of outliers with higher levels of homozygosity than the average for the genome (ROA would have more difficulty to detect such outliers if the phylogenetic inertia of adaptation is large). These scenarios are not mutually exclusive, and their contribution to lizards' local adaptation can be inferred with the outlier analyses described above. Our goal is not to identify the genetic basis of existing adaptations, but to unravel how evolution supplies the genetic variation that ecology demands for the convergence of phylogeographically divergent populations that are locally adapted to similar habitat.

## 2 MATERIALS AND METHODS

### 2.1 Field sampling

We sampled lizards in the focal population (Lerma) and in two pairs of populations that replicated either its eastern genetic lineage (Aranjuez and Brihuega of the E2 clade; Díaz et al., 2017; Verdú‐Ricoy, Iraeta, Salvador, & Díaz, 2014), or its forested habitat type within the distribution of the western lineage (El Pardo and Navacerrada of the W2 clade; Díaz et al., 2017). All these populations are located near the putative contact zone between the eastern and western lineages. Lerma (42.058°N, −3.611°E; 900 m asl) is a fragmented forest of evergreen and deciduous trees interspersed with grassland patches. Aranjuez (40.016°N, −3.586°E; 594 m asl) is a hot, dry site with a high cover of herbs and shrubs, and no trees, where all lizards are striped. Brihuega (40.778°N, −2.911°E; 1,009 m asl) is a deciduous open forest with a mosaic of grassland and woodland patches, where 67% of lizards are striped. El Pardo (40.511°N, −3.755°E; 658 m asl) is a xeric, lowland evergreen forest where 20% of lizards are striped, whereas Navacerrada (40.726°N, −4.023°E; 1,230 m asl) is a more productive montane location covered by deciduous forest where 17% of lizards are striped. Vegetation structure and species composition are very similar at Lerma and the western localities, especially Navacerrada; the three sites share a high cover of *Quercus* forests (deciduous *Q. pyrenaica* at Navacerrada and 66% of the Lerma forest fragments, and *Q. ilex* at El Pardo and 33% of the Lerma fragments) and a well‐developed layer of *Cistus* shrubs (*C. laurifolius* at Lerma and Navacerrada, and *C. ladanifer* at El Pardo).

### 2.2 Phenotypic variation

During the breeding season of 2012, we took tissue samples from 18 adult lizards (>1 year old) noosed or captured by hand in Lerma. During the breeding season of 2015, we captured and sampled with the same methods another 77 lizards (20, 18, 19, and 20 from Aranjuez, Brihuega, El Pardo, and Navacerrada, respectively). These 95 lizards were used for genomic analyses. In 2016, we captured 18 lizards in Lerma that were used for the analysis of phenotypes along with the 77 lizards captured in the other populations during 2015. Therefore, different individuals from Lerma were used for genetic and phenotypic analyses. In order to characterize lizards' phenotype, we brought them to the laboratory to record their snout‐vent length (SVL) and body mass and to measure their dorsal coloration from images (these are phenotypic measurements that have been suggested to be adaptive in previously published studies; Díaz et al., 2005, 2017; Iraeta, Salvador, & Díaz, 2013). For this later purpose, we held the individuals stretched under an antireflective glass and immobilized them against a soft sponge to avoid damaging them. We took pictures of the lizards' back in a dark room, using a fix setup with two white light sources placed on either side of the lizard at 25 cm from the subject, with an angle of 45°. The camera was set at 35 cm from the subject to obtain an overhead image, with automatic exposure compensation and shutter speed of 0.5 ms. We used Adobe Photoshop CS6 (Adobe Systems Incorporated, San Jose, CA, USA) for image processing (Llanos‐Garrido, Díaz, Pérez‐Rodríguez, & Arriero, 2017): We standardized the analyzed area using the “*magnetic lasso*” tool to delimit a 5‐cm‐long surface from the shoulder joint (which set the width of the analyzed surface) toward the posterior end of the animal. We measured the size of the striped surface with the “*magic wand*” tool (at 10% tolerance) after clicking at the middle of the stripe. We subsequently used the “*similar*” option of the “*magic wand*” tool (at 10% tolerance) to select pigmented scales and used the percentage of colored pixels in the analyzed surface as a measure of the size of the stripe. We measured the darkness of the stripe using the inverse of brightness of the colored layer. Note that we did not use a color standard to reduce the error in the measurement of brightness, but the fact that all lizards were photographed under the same conditions of illumination and exposure made our images directly comparable.

### 2.3 DNA extraction, sequencing, and variant calling

We obtained tissue samples by clipping 2 cm of the tail tip of lizards, which were released unharmed at their site of capture. We kept the samples in absolute ethanol at 4°C until DNA extraction. We purified DNA for library preparation using the Speedtools Tissue DNA Extraction Kit (Biotools) with a cell lysis step of 24 hr and resuspension in DNase‐free water at 60°C.

We used the restriction enzyme *Pst1* for GBS library preparation. Sequencing was done in an Illumina HiSeq2500 sequencer. The pipeline used for SNPs discovery was UNEAK, implemented in TASSEL v.3.0 (Bradbury et al., 2007), and specifically designed for samples with no reference genome. Sequence tags were aligned to each other to form “networks” of tags, where each node is a single tag sequence, and each edge represents a single base pair difference between two tags. The networks were pruned to remove putative sequencing errors (low frequency alleles) using the error rate threshold parameter. The resulting dataset had 83,648 SNPs with a site depth of 5.96 ± 6.56 (mean ± SD) and a site missingness of 0.49 ± 0.33. We discarded loci with minor allele frequencies <0.01 or that could be successfully sequenced in less than 10% of individuals. The resulting dataset had 73,291 loci, with only one SNP per locus, and with a site depth of 6.60 ± 6.75 and a site missingness of 0.42 ± 0.31.

### 2.4 Admixture and gene flow

Prior to performing clustering analyses, we used the software PLINK v.1.9. (Purcell et al., 2007) to prune the SNP database for linkage disequilibrium (LD), according to observed sample correlation coefficients. This was necessary because our clustering model (described below) did not take into account LD, and therefore, linked SNPs could bias the grouping. We performed clustering analyses to find genetic structure among populations and lineages (eastern or western) and to obtain the assignment probabilities of each individual to each one of the resulting clusters. We used the program ADMIXTURE v.1.3 (Alexander, Novembre, & Lange, 2009) for maximum‐likelihood estimation of individual ancestries. We used cross‐validation errors to identify the number of clusters (*K*‐value) for which the model had highest predictive accuracy. However, we also explored higher *K* values to detect patterns of genetic structure among the five populations. To complement such clustering analysis, we performed a multidimensional scaling analysis (MDS) on TASSEL 5.2. (Bradbury et al., 2007).

In order to improve the interpretation of our outlier analyses, we estimated gene flow among populations to test whether there could be less opposition to immigration of genetically western lizards in Lerma than in other populations of the eastern lineage. To obtain estimates of gene flow among populations, we used a maximum‐likelihood method based on coalescence implemented in MIGRATE 3.6 (Beerli & Felsenstein, 2001). MIGRATE uses an equilibrium model that estimates migration rates averaged across the coalescent history. Including undifferentiated populations in the analysis of migration rates would hamper the estimation of migration rates among less connected populations (Pfenninger & Posada, 2002). Therefore, we pooled individuals from populations that remained undifferentiated in an admixture analysis with *K* = 5 (equal to the number of sampled populations). Although grouping will make the estimates of local population size not interpretable for pooled populations, this strategy does provide robust estimates of gene flow (Beerli & Felsenstein, 2001). We used MIGRATE to estimate the effective population size scaled by mutation rate Θ = *N*_e_ *μ*, together with the effective number of migrants *N*_e_ *m*, where *N*_e_ is the effective population size, *μ* is the mutation rate per generation, and m is the migration rate per generation. Migration rates were estimated as *M* = *m*/*μ* and presented with 95% credibility intervals. Starting values for all parameter estimates were initially obtained using *F*_ST_ (Beerli & Felsenstein, 1999); all other searching parameters were set to default values. We performed a Markov chain Monte Carlo (MCMC) search running 10 heated chains with 100,000 recorded genealogies each, and three cold chains with 50,000 recorded genealogies, sampling each chain every 100 steps with a burn‐in period of 10,000 steps.

### 2.5 Outlier analyses

After estimating overall genetic structure and migration, we applied another filtering step to our SNP dataset to minimize false positives in the subsequent outlier analyses. To this end, we discarded loci with minor allele frequencies <0.05 in each population (thereby excluding all private alleles from the dataset), or loci that could not be successfully sequenced from at least 75% of individuals in each population. The resulting SNP dataset included 6,421 loci.

We used a Bayesian approach to perform a ROA as implemented in Bayescan v.2.1, a very useful approach when the number of populations is low (Foll & Gaggiotti, 2008). Bayescan uses a logistic regression model to partition *F*_ST_ coefficients into a population‐specific term (*β*) and a locus‐specific term (*α*). We selected loci with *α* > 0, suggesting positive selection and a conservative false discovery rate (corrected for multiple testing) of *q* ≤ 0.05. To obtain these parameters, we ran the reversible‐jump MCMC algorithm implemented in the program with a prior odd value of 10, and using 20 pilot runs of 5,000 iterations each, followed by 100,000 iterations with a burn‐in of 50,000. In order to search for outliers taking into account the variation in the degree of relatedness among populations, we performed a CIOA using the Bonhomme et al. (2010) extension of the Lewontin–Krakauer test, using R code following Bonhomme et al. (2010). This approach infers the historical branching of populations from the matrix of pairwise distances among populations, and identifies outliers based on *F*_ST_ deviations from the expected if fixation rates were proportional to the branch lengths of the population tree. We selected loci based on the statistical significance of the FLK statistic, with a restrictive significance threshold of *p* ≤ .001 to account for multiple testing. Finally, we used outliers inferred from ROA and CIOA to run new admixture analyses and compare them with the overall patterns of genetic admixture and gene flow. All the significance levels that we used in these analysis were as low as the lowest ones used in the demographic simulations performed by Bonhomme et al. (2010) in order to deal with false positives, which is a very common issue in genomic scans for traces of selection (Haasl & Payseur, 2016).

We analyzed variation among populations in the heterozygosity of outlier SNPs detected by ROA (*H*_ROA_) and CIOA (*H*_CIOA_), using ANCOVA with population as factor, heterozygosity of the relevant set of outliers as the dependent variable, and overall heterozygosity (*H*_ALL_) as the covariate.

## 3 RESULTS

### 3.1 Phenotypic variation

**Figure 2:**
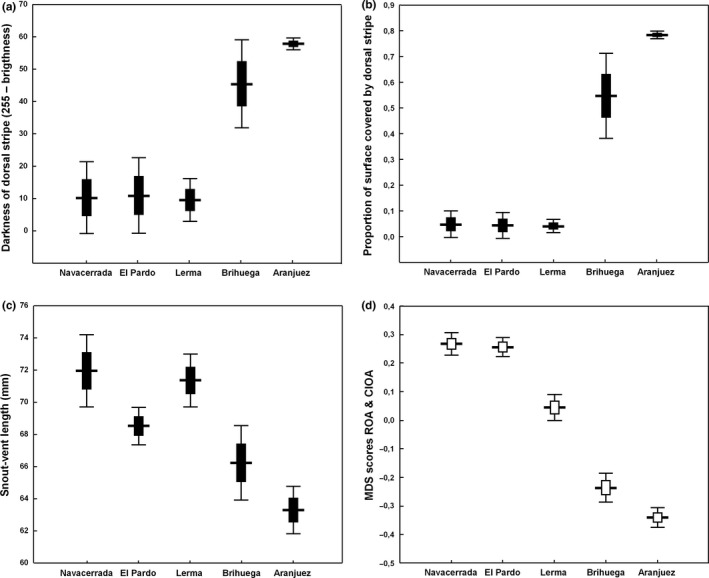
Phenotypic measures among populations (a–c) and MDS scores for outlier SNPs (d) (mean ± *SD* and 95% confidence interval). Population abbreviations: Aranjuez (A), Brihuega (B), Lerma (L), Navacerrada (N), El Pardo (P)

Phenotypic differences among populations were significant for all variables (ANOVAs for SVL, body mass, surface of the mid‐dorsal stripe, and darkness of the mid‐dorsal stripe: all *p* < 10^–6^; Figure 2a–c). Post hoc tests (Tukey's HSD for unequal *N*) revealed similar patterns for all phenotypes, with Aranjuez at one end of the phenotypic gradient and Lerma grouping with the western populations at the opposite end. Consistently with its suggested adaptive value, the proportion of striped lizards in each population remained strikingly constant between years, as shown by its high repeatability (intraclass correlation coefficient of 0.997) when comparing the values obtained in 2015 at Aranjuez, Brihuega, El Pardo, and Navacerrada with those reported by Díaz et al. (2017) for samples taken at the same sites between 2008 and 2012 (*r* ^2^ > .99, *n* = 4, *p* > .4 for the assumptions of the identity regression line that the slope and intercept do not differ significantly from one and zero, respectively). The proportion of striped lizards in Lerma (44%) was higher than in western populations, but their stripes were so tenuous that their overall phenotype was clearly western‐like (Figure 2a,b).

### 3.2 Admixture and gene flow

**Figure 3:**
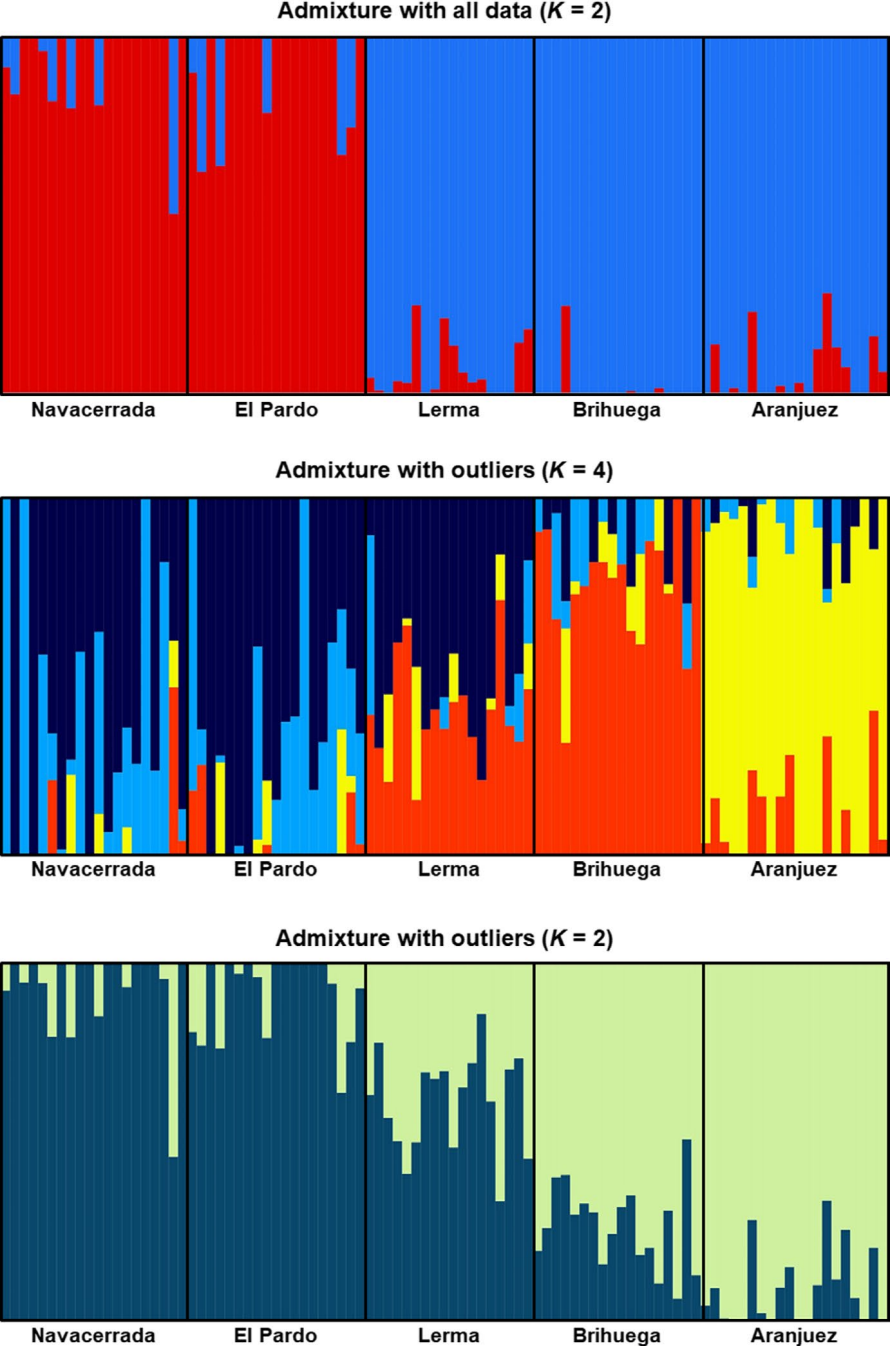
Genetic structure according to 73,291 SNPs (a) or 21 outlier SNPs (b, *K* = 4; c, *K* = 2). Bar colors represent posterior probabilities of cluster membership

**Figure 4:**
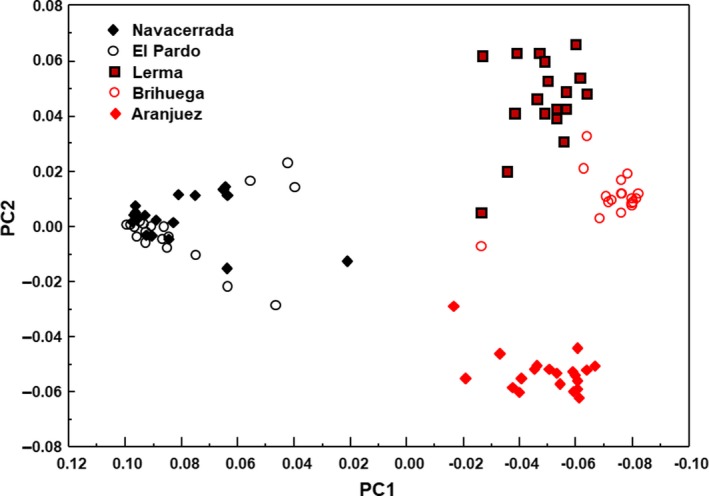
MDS analysis according to 73,291 SNPs that produced two major axes; the first of them consisted of a west–east gradient that discriminated between the two lineages, confirming the ancestral east–west division on a genome‐wide basis

Using 73,291 SNPs, the best model produced by ADMIXTURE had *K* = 2, recovering two clusters corresponding to the two mtDNA lineages (cv‐error = 0.586; Figure 3a). When we forced the model toward higher *K* values (with higher values of cv‐error), Aranjuez was the first population to separate from the rest of its lineage (*K* = 3, cv‐error = 0.665), followed by Brihuega and Lerma (*K* = 4, cv‐error = 0.744). However, when we set *K* equal to the number of populations in our sample, we did not detect any genetic structure between the two western populations (*K* = 5, cv‐error = 0.810). The MDS using the same dataset produced an axis with eigenvalue = 0.463, which defined an east–west gradient that discriminated between both mitochondrial lineages with *p* = 1.0 (*F*_1,93_ = 1,369.19, *R* ^2^ = .936, *p* < 10^–6^; mean ± *SD* scores of eastern and western lizards = −0.056 ± 0.017 and 0.081 ± 0.019, respectively; Figure 4).

**Figure 5:**
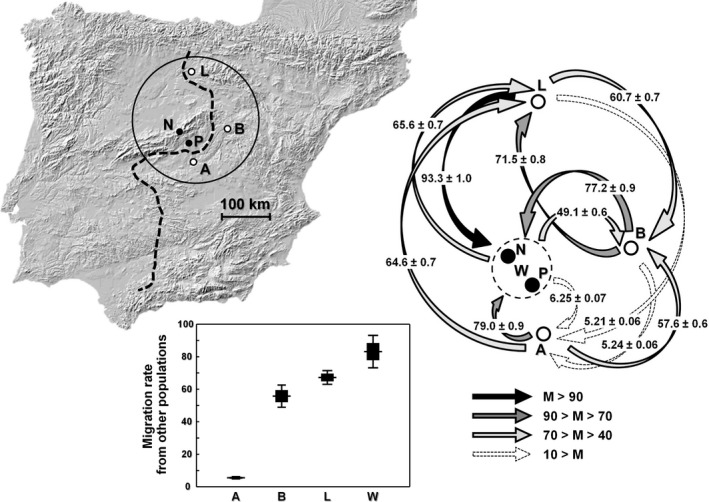
Location of the populations involved in the study (distributions of the two mtDNA lineages are divided by a solid black line) (a), maximum‐likelihood estimates of gene flow revealing asymmetrical patterns of migration among lizard populations (genetically undifferentiated western populations [W], i.e., Navacerrada and El Pardo, were pooled to perform this analysis and are represented with a dashed circle around them) (b), and immigration rate at each population, revealing genetic isolation in Aranjuez (c). Population abbreviations: Aranjuez (A), Brihuega (B), Lerma (L), Navacerrada (N), El Pardo (P). Open circles represent eastern populations, while closed circles represent western ones

Our maximum‐likelihood estimates of gene flow revealed highly asymmetrical patterns of migration among lizard populations (ANOVA for among‐group variation in the estimates of immigration: *F*_3,8_ = 105.10, *p* < .001; Figure 5). Among western lizards, MIGRATE recovered high levels of immigration from populations of the eastern lineage. Aranjuez was by far the most isolated population, while the other two eastern populations (Brihuega and Lerma) received intermediate levels of gene flow. Gene flow from western populations was higher in Lerma (Figure 5: *M* = 65.6 ± 0.7) than in Brihuega (49.1 ± 0.6) or Aranjuez (6.25 ± 0.07).

### 3.3 Outlier analyses

Our ROA with Bayescan detected 12 outlier loci with *α* > 0 (0.97 < *α* < 1.35) and *q* ≤ 0.05. The CIOA identified nine additional outlier loci with *p* < .001, none of which was detected by Bayescan. The MDS analysis performed with all 21 outliers yielded a single axis (eigenvalue = 6.78) representing a divergence gradient with western populations on one end and Aranjuez on the opposite end. This analysis placed Brihuega near Aranjuez, and Lerma close to the western populations, thus recovering the same pattern observed for lizard phenotypes (ANOVA with MDS scores: *F*_4,90_ = 180.98, *p* < .001; Figure 2d).

The best clustering model inferred by ADMIXURE using all the SNPs putatively under selection had *K* = 4 (cv‐error = 0.641), placing all individuals from Aranjuez and Brihuega into two clusters, and dividing individuals of Navacerrada and El Pardo into two clusters that did not match the western populations (Figure 3b). The population of Lerma was composed of individuals assigned either to one of the eastern clusters (the one including Brihuega, with a mean ± *SD* probability of assignment of 0.393 ± 0.147) or to one of the two western groups (probability of assignment of 0.547 ± 0.155). When we forced the model toward higher cv‐errors, the next result was for *K* = 2 (cv‐error = 0.686; Figure 3c), with all lizards being correctly assigned to their mitochondrial lineage except for those from Lerma that were assigned to the western cluster with mean ± *SD* probability of 0.613 ± 0.140.

Aranjuez and the two western populations, located at opposite extremes of the genetic gradient identified by MDS and admixture analyses, scored high homozygosity (with different alleles approaching fixation) for the outliers detected by ROA. Lerma, and to a lesser extent also Brihuega, scored significantly higher heterozygosity of ROA outliers than expected from overall genome heterozygosity (ANCOVA with population as the factor, *H*_ROA_ as the dependent variable and *H*_ALL_ as the covariate: *F*_4,89_ = 10.121, *p* < .001; Table 1). However, for the outliers detected by CIOA, Lerma scored significantly higher homozygosity than the other populations (*H*_CIOA_ as the dependent variable: *F*_4,89_ = 5.812, *p* < .001; Table 1). To further support the hypothesis that genetic variants subject to selection have been fixed along the evolutionary history of highly divergent populations (Aranjuez and western), we examined the probabilities of correct assignment of each individual to its lineage using SNP data with either whole genome data (PG, Figure 3a) or outliers (PO, Figure 3c). In the populations located at the extremes of the genetic gradient, we found a strong correlation between PO and PG (Aranjuez: *r* = .823, *p* < .001; western populations: *r* = .924, *p* < .001). However, the correlation was not significant in Brihuega (*r* = .294, *p* = .236) or in Lerma (*r* = .154, *p* = .541).

**Table 1:** Mean population values (±*SD*'s) for overall heterozygosity (*H*_ALL_), heterozygosity for outliers detected by ROA (*H*_ROA_), and heterozygosity for outliers detected by CIOA (*H*_CIOA_)

Population	*H*_ALL_	*H*_ROA_	*H*_CIOA_
Aranjuez (*N* = 20)	0.129 ± 0.035	0.209 ± 0.199 (0.191)	0.386 ± 0.270 (0.368)
Brihuega (*N* = 18)	0.119 ± 0.036	0.359 ± 0.158 (0.371)	0.247 ± 0.182 (0.260)
Lerma (*N* = 18)	0.136 ± 0.058	0.458 ± 0.237 (0.419)	0.183 ± 0.220 (0.144)
El Pardo (*N* = 19)	0.111 ± 0.043	0.178 ± 0.214 (0.212)	0.297 ± 0.200 (0.331)
Navacerrada (*N* = 20)	0.120 ± 0.041	0.131 ± 0.219 (0.139)	0.382 ± 0.199 (0.390)

Note

Adjusted means for *H*_ROA_ and *H*_CIOA_ (computed for the covariate, i.e., *H*_ALL_, at its mean) are shown in parentheses.

## 4 DISCUSSION

The large Psammodromus provides an example of local adaptation in which the phenotype of individuals fits better to the expected from habitat characteristics than from population ancestry. Our combined analysis of phenotypic variation, population genetic structure and genetic polymorphisms subject to selection, allowed us to explore the sources of genetic variants favored in different ecological contexts in this system.

The analysis of >73,000 SNPs recovered the east–west division of genetic lineages previously described on the basis of mitochondrial DNA sequences, thereby confirming the ancestral division of this species on a genome‐wide basis (Carranza et al., 2006). Compared to previous studies, our approach boosted the resolution with which the genetic background of lizard populations (and individuals) could be characterized. We found different degrees of population structure within genetic lineages, with hierarchical levels of between‐population differentiation within the eastern lineage (in which Aranjuez arose as an evolutionarily distinct population), and absence of differentiation between the two western populations studied (cf. Díaz et al., 2017). Within this genetic framework, our analysis of the dorsal striped versus unstriped pattern, a heritable trait with adaptive value in this species (Díaz et al., 2017), showed a phenotypic gradient with Aranjuez at one end, and the western populations at the other. The lizards from Lerma are phenotypically very similar to western lizards, a pattern that contradicts their unmistakably eastern genetic background, but is consistent with their western‐like habitat. This link between habitat and phenotype was in harmony with the pattern of genetic structuring of the outliers identified in the genome, an observation which substantiates the adaptive value of these genetic variants (Rhode et al., 2017; Rosenblum, Rompler, Schoneberg, & Hoekstra, 2010; Schield et al., 2017). Highly divergent populations (Aranjuez and western) have genetic variants subject to selection that seem to have been fixed along their evolutionary history. The high correlation between PG and PO in these populations further indicates that the position of individuals along the genetic gradient defined by selected alleles (outliers) corresponds to their location along the axis of genomic backgrounds opposing eastern and western lineages. In other words, it seems that the genetic variability that is adaptive in these populations (detected by ROA) is given by lineage sorting, and therefore, it could have been fixed along the long branches of the phylogenetic structure of our sample. However, the same correlation was not significant in Brihuega or in Lerma, suggesting that in these populations, selected alleles do not fit the overall pattern of divergence subject to selection (strongly determined by the splitting of eastern and western lineages, which seems to have arisen in an ecological scenario where Lerma hardly fits), either for the presence of new selected alleles (CIOA results in Lerma) or for high heterozygosity in alleles selected during lineage differentiation (ROA results in Brihuega and Lerma). Therefore, our results provide compelling evidence of ancestral genetic variation contributing to lizards' adaptability through its fixation along eastern and western lineages (Barrett & Schluter, 2008).

The process of local adaptation in Lerma seems to be favored not only by a genetic background consolidated through a long history of local adaptation to changing conditions, but also by the incorporation of genetic novelty (locally or imported from other populations; Barton & Etheridge, 2018). Gene flow among lizard populations was apparently influenced by ecological processes and not just by geographic proximity (Orsini, Vanoverbeke, Swillen, Mergeay, & De Meester, 2013; Rosenblum, 2006). For example, gene flow into ecologically extreme populations was asymmetric: Aranjuez was almost completely isolated while western populations scored the greatest levels of immigration. Aranjuez seems thus to be the place where incoming genotypes are more strongly purged out, probably because lizards occupy singular habitat of perhaps reduced quality. In forested habitat typical of western populations, the large Psammodromus prefers open forests where sunlit patches are readily available (Santos et al., 2008). In this habitat, genetically eastern lizards would not be selected against as strongly as the western ones may be purged out in shrubby habitat typical of eastern populations. In addition, eastern lizards may find opportunities in western habitat, where higher vegetation cover results into lower detectability by visually oriented predators (even with their striped phenotype; Díaz et al., 2017), and they can benefit from higher food abundance in forests (Consuegra, Verspoor, Knox, & García De Leániz, 2005; Tenan, Fasola, Volponi, & Tavecchia, 2017). The opposite is not true: Western lizards find poor habitat in open scrubland (such as Aranjuez in our study; Díaz et al., 2017). Also, eastern (striped) lizards could be better adapted to dispersal through unforested, grassy habitat than western (unstriped) ones (Duckworth, 2008; LaRue, Holland, & Emery, 2018). Whatever the causes of the asymmetry in migration rates found in our study, unrestricted gene flow from western populations, along with ancestral genetic variation, would allow eastern populations (especially Lerma) to approach the western lizard phenotype in forested habitat (Alleaume‐Benharira, Pen, & Ronce, 2006; Phillips, 1996).

Eastern and western lineages of the large Psammodromus have most likely remained locally adapted during the glacial cycles, maintaining their profound divergence but also great adaptive potential favored by the maintenance of ancestral genetic variation in the face of habitat heterogeneity (Barrett & Schluter, 2008; Gallet, Froissart, & Ravigné, 2018; Svardal, Rueffler, & Hermisson, 2015). Our outlier analysis (ROA) supports this idea, by documenting both the divergence of populations at the extremes of the environmental gradient, and the maintenance of high heterozygosity in eastern populations whose habitat has seemingly acquired western‐like conditions. In addition, admixture may also have been important for maintaining populations adapted to changing local conditions. Thus, a variable permeability to immigration conditioned by a dynamic landscape configuration may have prevented speciation while maintaining strong genetic structure (Räsänen & Hendry, 2008; Whitney, Bowen, & Karl, 2018). This is remarkable for a terrestrial vertebrate with limited dispersal ability (Santos et al., 2008) after 3 millions of years of lineage divergence (Carranza et al., 2006). At the same time, the combination of divergence subject to selection, maintenance of ancestral adaptive variation, and permeability to gene flow may have rendered the large *Psammodromus* a species able to adapt to a wide variety of habitats (Lande & Shannon, 2006; Mackay, 1981; Paccard et al., 2018; Parter et al., 2008; Peniston et al., 2019; Welch & Jiggins, 2014), a trait which may underlie its success as the most abundant and widespread Iberian lizard (Huang et al., 2016; Pleguezuelos, Marquez, & Lizana, 2004).

However, an obvious question is why new genetic variants (CIOA) have evolved in Lerma when this population already had the ancestral genetic variation (at ROA loci highly homozygous in the west) that could be selected to warrant local adaptation to western‐like habitat. This paradox may hide a more complex scenario in which environmental gradients are multidimensional, with other factors (such as predators, parasites, or competitors) affecting the dynamics of local adaptation besides the forest‐to‐open gradient driving the divergence between the two lineages (Aguirre‐Liguori et al., 2017; Levinst, 1962). After all, if only one dimension of environmental variation is considered, moving away from one end of the gradient inevitably leads to approaching the other end (Lahti et al., 2009).

All in all, the combined use of ROA and CIOA provided compelling evidence of different sources of genetic variation contributing to local adaptation dynamics in a lizard species. We are aware that this was feasible thanks to various singularities of our study system, with populations distributed over a wide, heterogeneous range that has been changing since the split of eastern and western lineages in the late Pliocene. In this biogeographical scenario, adaptive lineage splitting was counterbalanced by ancestral genetic variation and asymmetrical patterns of gene flow (likely promoted by historical or current environmental instability), which could hamper speciation despite strong genetic structure. Complex systems like this one may thus exemplify how to shed light on a variety of important questions at the interface between local adaptation and the first steps toward speciation (or the lack of it; Rosenblum, 2006; Rundle & Nosil, 2005; Schluter, 2001; Via, 2009). Overall, the combined use of ROA and CIOA may offer a powerful tool to elucidate how evolution provides the genetic variation that ecology demands throughout the history of a species.

## CONFLICT OF INTEREST

None declared.

## AUTHOR CONTRIBUTIONS

AL‐G, JP‐T, and JAD initiated and designed the study; AL‐G and JAD undertook fieldwork; AL‐G conducted molecular work; AL‐G analyzed molecular data; AL‐G, JP‐T, and JAD analyzed nonmolecular data; AL‐G wrote the first draft, and JP‐T and JAD contributed substantially with the revisions that led to the final manuscript.

## Data Availability

Data for this study are available at PANGAEA (Llanos‐Garrido, Pérez‐Tris, & Díaz, 2019): https://doi.org/10.1594/PANGAEA.908220.
